# Mammographic density and risk of breast cancer according to tumor characteristics and mode of detection: a Spanish population-based case-control study

**DOI:** 10.1186/bcr3380

**Published:** 2013-01-29

**Authors:** Marina Pollán, Nieves Ascunce, María Ederra, Alberto Murillo, Nieves Erdozáin, Jose Enrique Alés-Martínez, Roberto Pastor-Barriuso

**Affiliations:** 1 National Center for Epidemiology, Carlos III Institute of Health, Monforte de Lemos 5, Madrid, 28029 Spain; 2 Consortium for Biomedical Research in Epidemiology and Public Health (CIBER en Epidemiología y Salud Pública-CIBERESP), Carlos III Institute of Health, Monforte de Lemos 5, Madrid, 28029, Spain; 3 Navarre Breast cancer Screening Program, Navarre Institute of Public Health, Leyre 15, Pamplona, 31003, Spain; 4 Medical Oncology Unit, Nuestra Señora de Sonsoles Hospital, Avenida Juan Carlos I s/n, Avila, 05004, Spain

## Abstract

**Introduction:**

It is not clear whether high mammographic density (MD) is equally associated with all subtypes of breast cancer (BC). We investigated the association between MD and subsequent BC, considering invasiveness, means of detection, pathologic subtype, and the time elapsed since mammographic exploration and BC diagnosis.

**Methods:**

BC cases occurring in the population of women who attended screening from 1997 through 2004 in Navarre, a Spanish region with a fully consolidated screening program, were identified via record linkage with the Navarre Cancer Registry (*n = *1,172). Information was extracted from the records of their first attendance at screening in that period. For each case, we randomly selected four controls, matched by screening round, year of birth, and place of residence. Cases were classified according to invasiveness (ductal carcinoma *in situ *(DCIS) versus invasive tumors), pathologic subtype (considering hormonal receptors and HER2), and type of diagnosis (screen-detected versus interval cases). MD was evaluated by a single, experienced radiologist by using a semiquantitative scale. Data on BC risk factors were obtained by the screening program in the corresponding round. The association between MD and tumor subtype was assessed by using conditional logistic regression.

**Results:**

MD was clearly associated with subsequent BC. The odds ratio (OR) for the highest MD category (MD >75%) compared with the reference category (MD <10%) was similar for DCIS (OR = 3.47; 95% CI = 1.46 to 8.27) and invasive tumors (OR = 2.95; 95% CI = 2.01 to 4.35). The excess risk was particularly high for interval cases (OR = 7.72; 95% CI = 4.02 to 14.81) in comparison with screened detected tumors (OR = 2.17; 95% CI = 1.40 to 3.36). Sensitivity analyses excluding interval cases diagnosed in the first year after MD assessment or immediately after an early recall to screening yielded similar results. No differences were seen regarding pathologic subtypes. The excess risk associated with MD persisted for at least 7 to 8 years after mammographic exploration.

**Conclusions:**

Our results confirm that MD is an important risk factor for all types of breast cancer. High breast density strongly increases the risk of developing an interval tumor, and this excess risk is not completely explained by a possible masking effect.

## Introduction

In Spain, a European country with moderate incidence of breast cancer (BC), all the Autonomous Regions introduced population-based BC screening programs during the 1990s, with full coverage being attained by the beginning of this century [1]. Although regional differences exist, these programs have been well received, and participation rates are usually high (mean overall participation of 67%) [1]. Indeed, screening has had a clear influence on Spanish BC trends [2]. Currently, all Spanish women aged 50 to 69 or 45 to 69 years, depending on the region, are invited to have a mammogram every other year, amounting to a total catchment population of more than 5 million.

Mammograms are useful not only from a diagnostic point of view. Mammographic density (MD), or the percentage of the mammogram occupied by radiologically dense tissue, is a well-established BC risk factor [3]. MD is highly heritable [4] but is also influenced by classic BC determinants, such as parity, benign breast disease, and combined hormonal therapy [5-7]. Even though the biologic basis of the association between MD and BC is not fully understood, breast density is increasingly used as a phenotype risk marker [5].

At present, screening guidelines, both in Europe and in the United States, recommend mammography every 2 years [8,9]. In practice, family history and benign pathology of the breast are taken into account to recommend early recalls. A recent study proposed the inclusion of MD in a personalized scheme for screening follow-up [10]. Before incorporating MD assessment into the decision algorithm for cancer prevention, it is essential to confirm its ability to predict all BC subtypes, and more-aggressive tumors in particular, something that has not been fully established [5]. Breast density impairs tumor detection, increasing the probability of false-negative results [11,12]. As regards tumor subtypes, whereas some cohort and case-control studies have examined the association between high MD and BC according to type of tumor, many of these included a low number of cases, and their results are not homogeneous [13-19].

This article relies on data from a population-based case-control study conducted in the context of the Navarre Breast Cancer Screening Program (NBCSP) to evaluate the association between MD and subsequent risk of BC, according to tumor invasiveness, pathologic subtype, and means of detection.

## Materials and methods

### Study population

The NBCSP, the first population-based screening program implemented in Spain, was initiated in 1990 and achieved full coverage by 1992 [1]. Whereas the NBCSP initially targeted women aged 45 to 65 years residing in the Northern Spanish province of Navarre, in 1998, the age range was extended to include women aged up to 69 years (77,455 female inhabitants aged 45 to 69 years in 2001). In general, women are screened every 2 years, following European guideline recommendations [8]. The overall participation rate and adherence to the NBCSP during the period from 1990 to 2004 were very high (88% and 97%, respectively). A detailed description of the program can be consulted elsewhere [20].

The current case-control study is based on the population of women who attended the NBCSP at least once between the fourth (September 1996 to August 1998) and seventh (September 2002 to August 2004) screening rounds. These women were prospectively followed up for BC occurrence from the date of their first NBCSP scan in the study period, until December 31, 2005. BC cases, whether invasive tumors or ductal carcinomas *in situ *(DCISs), were ascertained through linkage with the population-based Navarre Cancer Registry. To ensure completeness, follow-up was restricted to year-end 2005, the last complete year available when the study was designed. During follow-up, in total, 3,003 new BC cases were registered among women living in Navarre: 2,259 occurred in women who had participated in the screening program, and 1,461 corresponded to women who had attended screening at least once between the fourth and seventh rounds. Cases diagnosed during the first 6 months after their first contact with the NBCSP between the fourth and seventh rounds (baseline screening round) were considered prevalent and therefore excluded (*n *= 289). For the remaining 1,172 incident cases, the average time elapsed from baseline to date of BC diagnosis was 4.3 years (interquartile range, 2.1 to 5.9 years).

### Case and control selection

We included all 1,172 incident cases of invasive BC or DCIS diagnosed in the period between 6 months after baseline (first NBCSP attendance between the fourth and the seventh rounds) and December 31, 2005. All cases were classified on the basis of the means of detection as screen-detected cases, interval cases, or external cases, with the latter group comprising cases diagnosed in women older than 69 years (NBCSP upper age limit) plus those registered in women who had prematurely withdrawn from the program. Based on pathology reports, invasive BC cases were further classified as estrogen and/or progesterone receptor-positive and HER2-negative tumors, HER2-positive tumors, or triple-negative tumors.

In total, 4,688 controls were randomly selected (case-to-control ratio, 1:4) from all women in the source population who remained disease free at the end of follow-up. Controls were frequently matched to cases by screening round, single year of birth, and place of residence (single towns with more than 5,000 inhabitants or aggregated rural areas comprising smaller towns).

### Mammographic density and other risk factors

Information for cases and their matched controls was obtained from mammograms and epidemiologic questionnaires collected by the NBCSP at the baseline screening round, defined as the first screening attendance of the corresponding case between the fourth (September 1996 to August 1998) and the seventh (September 2002 to August 2004) rounds of the NBCSP. Breast density was assessed from the mediolateral oblique mammographic projection of the left breast. This was chosen because it was the only projection available during the fourth NBCSP round for previous participants. MD was visually assessed by a single, experienced radiologist blinded to case/control status, by using the Boyd semiquantitative scale with the following categories of density: 0; <10%; 10% to 25%; 25% to 50%; 50% to 75%; and >75%. The radiologist had been previously trained in using a transparent grid template to quantify the total breast area and the area composed of dense tissue. After the training process, mediolateral oblique-view mammograms from 100 randomly selected disease-free women were read twice in different random orders to assess intrarater reliability, which proved to be substantially high (quadratic-weighted kappa: 0.93; 95% CI = 0.89 to 0.96). Very good agreement was found between MD measurements drawn from mediolateral oblique and craniocaudal mammographic projections in the same random sample of 100 women (quadratic-weighted kappa, 0.92; 95% CI = 0.88 to 0.94). Because of the small number of women with a MD of 0, the first two categories were collapsed and used in all analysis as the reference group (MD = 0 to 10%).

Baseline information on age at menarche, parity, age at first live birth, menopausal status, age at menopause, number of first-degree and second-degree relatives with BC, age of affected relatives at diagnosis, previous breast biopsy, and current use of hormone replacement therapy was obtained from structured questionnaires administered by trained interviewers.

### Statistical analysis

Because the number of cases in each stratum obtained by cross-classifying the three matching factors was typically sparse, the association between MD plus other baseline explanatory variables and BC risk was evaluated by using conditional logistic regression models to account fully for the matched structure of the data. Crude odds ratios (adjusted solely for the matching variables) and adjusted odds ratios (considering all risk factors associated with BC in crude analyses) were obtained. Because of the small number of women with low density, the two first categories of MD were combined and used as the reference group. The multivariate model was separately fitted for DCIS and invasive tumors. The same model was used to assess the association between MD and other explanatory variables, and screen-detected and interval cases. The number of cases detected among women who had withdrawn from the program was too small for these to be deemed a separate group.

The stability of the association between MD and BC was explored, taking into account the time span between the date of baseline exploration (that is, the moment when MD and the remaining variables were assessed) and the date of diagnosis, considering three periods (that is, less than 3 years, 3 to 6 years, and 6 years and longer). Furthermore, to test the consistency of the excess risk associated with high MD, subgroup analyses were conducted by including interaction terms for MD density (four categories: 0 to 10%, 10% to 25%, 25% to 50%, and >50%) with the other explanatory variables.

Finally, with the information provided by pathology records, the final model was separately fitted for each pathologic subtype, considering the previously mentioned four categories of MD. To test whether the association differed by pathologic subtype, a multinomial logistic model was used, adjusting for screening round, age at screening, and the remaining variables. The likelihood ratio test was used to compare a model with separate MD slopes for each tumor type with a model constrained to have a common slope [21].

All statistical analyses were performed by using the STATA version 12.0 software program (Stata Corp, College Station, TX, USA).

### Ethical considerations

The study was approved by the Ethics Committee of the Carlos III Institute of Health (Comité de Ética de la Investigación y De Bienestar Animal CEIyBA ISCIII) and conducted in compliance with the Helsinki Declaration. Specific patient informed consent was not required for this study, because all women consented to participate in the NBCSP, and the program was authorized to collect and use health and clinical information from screening participants for evaluation and scientific research.

## Results

The study included 1,172 incident breast cancer cases, made up of 184 DCIS and 988 invasive tumors, and 4,688 matched controls. Seventy-five percent of cases were diagnosed directly by the NBCSP (875), 251 (21%) were interval cases, and only 46 (4%) were observed in women who had withdrawn from the program. In most instances, baseline exploration corresponded to the fourth round (84%). Average age at exploration was 53 years. Around one third of these women were premenopausal (Table 1). Compared with controls, cases had a higher proportion of nulliparous women (19% versus 13%), family history of BC (19% versus 14%), particularly in terms of first-degree relatives (10% versus 6%), and a higher frequency of previous biopsies (15% versus 9%). However, use of hormonal replacement treatment was similar in both groups (11% versus 10%). MD was higher among cases, with 23% of them being in the two highest categories (MD >50%), as opposed to 14% of controls. At the other extreme, 37% of controls had less than 10% of dense tissue versus 22% among cases (Table 1).

**Table 1:** Baseline characteristics of cases and controls, and odds ratios for total breast cancer in the NBCSP case-control study

Characteristic	No. of controls (%)	No. of breast cancer cases (%)	Odds ratio^a^(95% CI)	*P *value^b^
Screening round (baseline exploration)				
September 1996 to August 1998	3,948 (84.1)	987 (84.1)		
September 1998 to August 2000	408 (8.7)	102 (8.7)		
September 2000 to August 2002	224 (4.8)	56 (4.8)		
September 2002 to August 2004	108 (2.3)	27 (2.3)		
Age at baseline exploration (years)				
<50	1,733 (37.0)	433 (37.0)		
50-54	986 (21.0)	245 (20.9)		
55-59	897 (19.1)	228 (19.5)		
60-64	870 (18.6)	217 (18.5)		
≥65	202 (4.3)	49 (4.2)		
Age at menarche (years)				0.357^c^
≥15	724 (15.5)	161 (13.7)	0.95 (0.77-1.18)	
14	1,243 (26.5)	290 (24.7)	1.00 (reference)	
13	1,281 (27.3)	363 (31.0)	1.22 (1.03-1.45)	
12	867 (18.5)	225 (19.2)	1.11 (0.92-1.35)	
<12	569 (12.1)	132 (11.3)	1.00 (0.79-1.25)	
Unknown	4 (0.1)	1 (0.1)		
Age at first live birth (years)				<0.001
<20	102 (2.2)	13 (1.1)	0.51 (0.28-0.91)	
20-24	1,441 (30.7)	293 (25.0)	0.81 (0.68-0.95)	
25-29	1,823 (38.9)	457 (39.0)	1.00 (reference)	
30-34	522 (11.1)	134 (11.4)	1.03 (0.83-1.28)	
≥35	175 (3.7)	57 (4.9)	1.31 (0.95-1.80)	
Nulliparous	625 (13.3)	218 (18.6)	1.41 (1.17-1.70)	
Age at menopause (years)				0.001
≤45	777 (16.6)	147 (12.5)	1.00 (reference)	
46-50	1,244 (26.5)	298 (25.4)	1.25 (1.01-1.56)	
>50	1,035 (22.1)	293 (25.0)	1.48 (1.17-1.87)	
Premenopausal	1,631 (34.8)	434 (37.0)	1.44 (1.14-1.83)	
Unknown	1 (0.0)	0 (0.0)		
Family history of breast cancer				<0.001
None	4,075 (86.9)	945 (80.6)	1.00 (reference)	
Second-degree relative	327 (7.0)	109 (9.3)	1.43 (1.14-1.80)	
First-degree relative ≥50 years	172 (3.7)	64 (5.5)	1.61 (1.19-2.16)	
First-degree relative <50 years	114 (2.4)	54 (4.6)	2.06 (1.48-2.88)	
Previous breast biopsy				<0.001
No	4,281 (91.3)	1,001 (85.4)	1.00 (reference)	
Yes	407 (8.7)	171 (14.6)	1.82 (1.50-2.22)	
Use of hormone replacement therapy				0.602
No	4,208 (89.8)	1,046 (89.2)	1.00 (reference)	
Yes	480 (10.2)	126 (10.8)	1.06 (0.86-1.31)	
Mammographic density (%)				<0.001^c^
0-10	1,711 (36.5)	263 (22.4)	1.00 (reference)	
11-25	1,136 (24.3)	244 (20.8)	1.52 (1.25-1.85)	
26-50	1,158 (24.8)	375 (32.0)	2.47 (2.05-2.98)	
51-75	529 (11.3)	215 (18.3)	3.27 (2.62-4.09)	
>75	135 (2.9)	59 (5.0)	3.74 (2.63-5.32)	
Unknown	19 (0.4)	16 (1.4)		

^a^Odds ratios for total breast cancer and 95% confidence intervals (CIs) obtained from conditional logistic regression models that accounted for the matching factors (screening round, year of birth, and place of residence). ^b^*P *values for homogeneity of breast cancer risk across categories, except in the case of age at menarche and mammographic density. ^c^*P *values for linear trend using an ordinal variable

Table 1 also shows crude odds ratios (ORs) and 95% confidence intervals (95% CIs) for the explanatory variables. Linear trend tests were obtained, including the categoric variable as a continuous term. Most of the risk factors considered were associated with breast cancer. However, neither age at menarche nor use of hormonal replacement therapy at the date of exploration modified BC risk in our study (Table 1). As regards family history of BC, a distinction was drawn among first-degree relatives according to their age at diagnosis, taking age 50 years as cutoff. The highest risk was observed among women with first-degree relatives aged younger than 50 years at diagnosis (OR = 2.06; 95% CI = 1.48 to 2.88). MD, our exposure of interest, showed a clear positive association with BC (*P-*trend <0.001). The two highest categories of density (>50%) registered ORs of >3, compared with the reference category (<10%) (OR = 3.27; 95% CI = 2.62 to 4.09 for MD = 50% to 75%; and OR = 3.74; 95% CI = 2.63 to 5.32 for MD >75%).

Table 2 shows the results of the multivariate analysis both overall, for all cases combined, and for cases stratified by tumor invasiveness (DCIS or invasive cancer). Adjusting for other risk factors had a modest effect on the association between MD and BC. ORs were similar for DCIS and invasive tumors (OR_DCIS _= 3.47; 95% CI = 1.46 to 8.27; and OR_invasive _= 2.78; 95% CI = 1.87 to 4.06 for MD >75%), and a clear dose-response with increasing MD was observed in both instances. Table 3 shows the results obtained with the same multivariate model for screen-detected BC cases and interval tumors. ORs were particularly high in the two highest categories of density for interval tumors (OR = 4.25; 95% CI = 2.53 to 7.14, and OR = 7.72l; 95% CI = 4.02 to 14.81 for MD of 50% to 75% and >75%, respectively). To avoid a possible masking effect of MD, which might produce a false-negative result at screening, the analysis was repeated by taking only case-control sets with interval cases diagnosed more than 12 months after mammographic assessment (225 cases). The results were very similar (OR = 3.93; 95% CI = 2.26 to 6.82 for the 50% to 75% category, and OR = 7.62; 95% CI = 3.82 to 15.19 for MD >75%). Furthermore, the exclusion of interval cases observed after an exploration that was motivated by an early recall (21 cases), yielded similar results (OR = 4.22; 95% CI = 2.47 to 7.21 for MD 50% to 75%; and OR = 7.93; 95% CI = 4.00 to 15.70 for MD >75%).

**Table 2:** Association between mammographic density and other selected risk factors, and risk of total, invasive, and *in situ *breast cancer in the NBCSP case-control study

		Total breast cancer			Invasive breast cancer			Ductal carcinoma *in situ*	
						
Baseline risk factor	No. of controls (%)	No. of cases (%)	Odds ratio^a^ (95% CI)		No. of cases (%)	Odds ratio^a^ (95% CI)		No. of cases (%)	Odds ratio^a^ (95% CI)
Age at first live birth^b^									
5-year increase	4,048 (86.8)	940 (81.3)	1.12 (1.02-1.22)		795 (81.8)	1.10 (1.00-1.21)		145 (78.8)	1.19 (0.97-1.46)
Nulliparous	618 (13.2)	216 (18.7)	1.34 (1.12-1.61)		177 (18.2)	1.30 (1.06-1.58)		39 (21.2)	1.58 (1.04-2.40)
Age at menopause^c^									
5-year increase	3,040 (65.2)	728 (63.0)	1.18 (1.06-1.30)		619 (63.7)	1.17 (1.05-1.30)		109 (59.2)	1.20 (0.92-1.55)
Premenopausal	1,626 (34.8)	428 (37.0)	1.14 (0.93-1.41)		353 (36.3)	1.20 (0.95-1.51)		75 (40.8)	0.96 (0.59-1.55)
Family history of breast cancer									
None	4,055 (86.9)	932 (80.6)	1.00 (reference)		790 (81.3)	1.00 (reference)		142 (77.2)	1.00 (reference)
Second-degree relative	325 (7.0)	107 (9.3)	1.39 (1.10-1.75)		89 (9.2)	1.39 (1.08-1.79)		18 (9.8)	1.42 (0.83-2.43)
First-degree relative ≥50 years	172 (3.7)	64 (5.5)	1.46 (1.08-1.98)		49 (5.0)	1.33 (0.95-1.87)		15 (8.2)	2.11 (1.13-3.94)
First-degree relative <50 years	114 (2.4)	53 (4.6)	1.90 (1.35-2.68)		44 (4.5)	2.00 (1.38-2.91)		9 (4.9)	1.63 (0.77-3.47)
Previous breast biopsy									
No	4,263 (91.4)	986 (85.3)	1.00 (reference)		826 (85.0)	1.00 (reference)		160 (87.0)	1.00 (reference)
Yes	403 (8.6)	170 (14.7)	1.57 (1.28-1.92)		146 (15.0)	1.68 (1.36-2.08)		24 (13.0)	1.11 (0.68-1.83)
Mammographic density (%)									
0-10	1,710 (36.6)	263 (22.7)	1.00 (reference)		233 (24.0)	1.00 (reference)		30 (16.3)	1.00 (reference)
11-25	1,136 (24.3)	244 (21.1)	1.43 (1.18-1.74)		206 (21.2)	1.39 (1.12-1.71)		38 (20.7)	1.65 (1.01-2.83)
26-50	1,157 (24.8)	375 (32.4)	2.24 (1.85-2.71)		319 (32.8)	2.18 (1.78-2.68)		56 (30.4)	2.65 (1.60-4.40)
51-75	528 (11.3)	215 (18.6)	2.76 (2.19-3.48)		164 (16.9)	2.38 (1.85-3.06)		51 (27.7)	5.60 (3.24-9.67)
>75	135 (2.9)	59 (5.1)	3.06 (2.14-4.40)		50 (5.1)	2.95 (2.01-4.35)		9 (4.9)	3.47 (1.46-8.27)
*P *value for trend^d^			<0.001			<0.001			< 0.001

^a^Odds ratios and 95% confidence intervals (CIs) for total, invasive, and *in situ *breast cancer obtained from separate multivariate conditional logistic regression models adjusted for all risk factors shown in the table. ^b^Adjusted odds ratios per 5-year increase in age at first live birth among parous women, as well as for nulliparous women compared with women having their first live birth at 25 years. ^c^Adjusted odds ratios per 5-year increase in age at menopause among postmenopausal women, as well as for premenopausal women compared with women having their menopause at 45 years. ^d^*P *values for linear trend by using an ordinal variable with values 1 through 6 across successive categories of mammographic density.

**Table 3:** Association between mammographic density and other selected risk factors, and risk of total breast cancer stratified by means of detection in the NBCSP case-control study

		Screen-detected breast cancer			Interval breast cancer	
				
Baseline risk factor	No. of controls (%)	No. of cases (%)	Odds ratio^a^(95% CI)		No. of cases (%)	Odds ratio^a^(95% CI)
Age at first live birth^b^						
5-year increase	4,048 (86.8)	705 (81.0)	1.11 (1.00-1.23)		202 (84.2)	1.17 (0.98-1.41)
Nulliparous	618 (13.2)	165 (19.0)	1.38 (1.12-1.70)		38 (15.8)	1.14 (0.77-1.70)
Age at menopause^c^						
5-year increase	3,040 (65.2)	573 (65.9)	1.17 (1.05-1.31)		121 (50.4)	1.18 (0.92-1.50)
Premenopausal	1,626 (34.8)	297 (34.1)	1.04 (0.82-1.32)		119 (49.6)	1.56 (1.02-2.38)
Family history of breast cancer						
None	4,055 (86.9)	712 (81.8)	1.00 (reference)		184 (76.7)	1.00 (reference)
Second-degree relative	325 (7.0)	75 (8.6)	1.24 (0.95-1.63)		28 (11.7)	1.83 (1.16-2.87)
First-degree relative ≥50 years	172 (3.7)	49 (5.6)	1.49 (1.06-2.10)		13 (5.4)	1.55 (0.80-2.97)
First-degree relative <50 years	114 (2.4)	34 (3.9)	1.57 (1.05-2.36)		15 (6.3)	2.83 (1.53-5.22)
Previous breast biopsy						
No	4,263 (91.4)	747 (85.9)	1.00 (reference)		200 (83.3)	1.00 (reference)
Yes	403 (8.6)	123 (14.1)	1.48 (1.18-1.86)		40 (16.7)	1.99 (1.34-2.96)
Mammographic density (%)						
0-10	1,710 (36.6)	220 (25.3)	1.00 (reference)		32 (13.3)	1.00 (reference)
11-25	1,136 (24.3)	188 (21.6)	1.30 (1.05-1.62)		46 (19.2)	1.95 (1.20-3.17)
26-50	1,157 (24.8)	273 (31.4)	2.00 (1.62-2.47)		87 (36.3)	3.54 (2.24-5.60)
51-75	528 (11.3)	155 (17.8)	2.45 (1.89-3.17)		52 (21.7)	4.25 (2.53-7.14)
>75	135 (2.9)	34 (3.9)	2.17 (1.40-3.36)		23 (9.6)	7.72 (4.02-14.81)
*P *value for trend^d^			<0.001			< 0.001

^a^Odds ratios and 95% confidence intervals (CIs) for screen-detected and interval breast cancer obtained from separate multivariate conditional logistic regression models adjusted for all risk factors shown in the table. ^b^Adjusted odds ratios per 5-year increase in age at first live birth among parous women, as well as for nulliparous women compared with women having their first live birth at 25 years. ^c^Adjusted odds ratios per 5-year increase in age at menopause among postmenopausal women, as well as for premenopausal women compared with women having their menopause at 45 years. ^d^*P *values for linear trend by using an ordinal variable with values 1 through 6 across successive categories of mammographic density.

**Figure 1:**
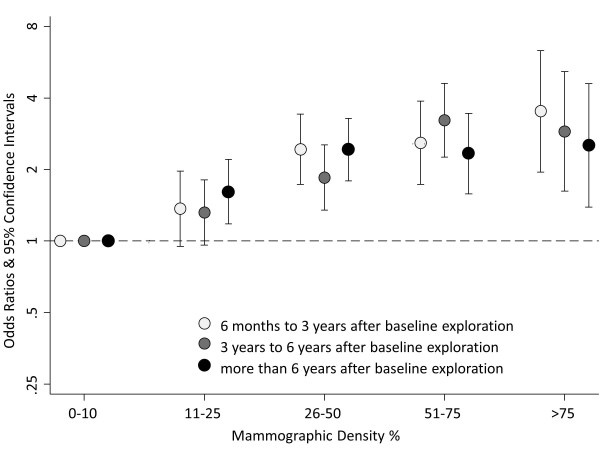
**Association between mammographic density and breast cancer according to time elapsed between baseline screening exploration and tumor diagnosis**. Odds ratios and 95% confidence intervals adjusted for age, age at first live birth, menopausal status and age at menopause, familial history of breast cancer, and history of previous biopsies.

The excess risk associated with higher density appeared to remain stable for at least the first 7 to 8 years after mammographic assessment (Figure 1). The relative linear increase in risk per category of density was similar in the three groups considered (OR = 1.40; 95% CI = 1.25 to 1.56 for tumors diagnosed 6 months to 3 years after exploration; OR = 1.41; 95% CI = 1.27 to 1.56 for those diagnosed between 3 and 6 years after exploration; and OR = 1.33; 95% CI = 1.20 to 1.48 for cancers detected 6 or more years after exploration).

**Figure 2:**
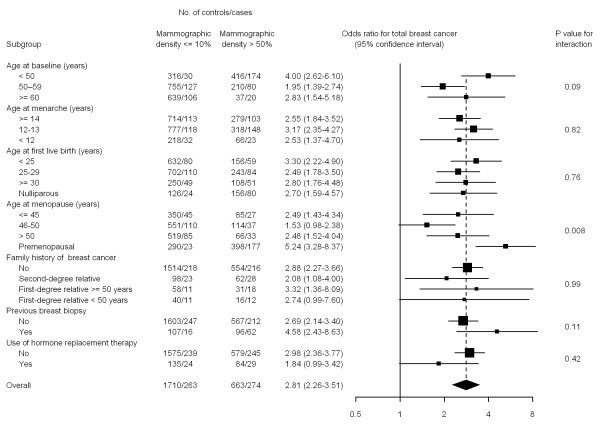
**Association between mammographic density (MD) and breast cancer**. Odds ratios and 95% confidence intervals for MD >50% compared with MD = 0 to 10% per category of other explanatory variables. Estimators are adjusted for age, age at first live-birth, menopausal status and age at menopause, familial history of breast cancer, and history of previous biopsies.

Figure 2 depicts the effect of high MD per category of other explanatory variables. Given the small number of subjects in some strata, the two extreme categories of density were collapsed at both sides. The figure shows the ORs for the highest MD (>50%) versus the reference category (0 to 10%), adjusted for the factors included in the multivariate model (Tables 2 and 3). With few exceptions, the effect was very consistent across all strata. The association was stronger in younger women and particularly among those who were premenopausal at the date of exploration (*P *value for interaction = 0.008). Given this different effect in pre-and postmenopausal women, the final model was separately fitted in these two groups (see Additional file 1, Table S1). In postmenopausal women, the ORs for the two highest MD categories were 2.10 (95%CI = 1.52 to 2.89) for a density of 50% to 75% and 1.98 (95% CI = 1.04 to 3.76) for MD >75%. The corresponding estimators in premenopausal women were 4.88 (95% CI = 2.97 to 8.01) and 5.13 (95% CI = 2.83 to 9.30).

Information on hormonal receptors and HER2 status was available for 86% of invasive cases (*N *= 834) but not for a substantial proportion of DCIS. Table 4 shows the effect of MD and the remaining explanatory variables included in the final model, according to pathologic subtype of invasive BC. MD was similarly associated with all pathologic subtypes (*P *value of heterogeneity = 0.380), although ORs were somewhat higher for triple-negative tumors (for MD >50% versus MD = 0 to 10%: OR = 3.15; 95% CI = 1.26 to 7.84), intermediate for tumors with hormonal receptors and no expression of HER2 (OR = 2.60; 95% CI = 1.95 to 3.45), and weaker for HER2^+ ^tumors (OR = 1.68; 95% CI = 0.83 to 3.41). This analysis was repeated by subdividing HER2^+ ^tumors according to the presence/absence of hormonal receptors (65 and 46 cases, respectively), with similar results being obtained in both groups (data not shown).

**Table 4:** Association between mammographic density and other selected risk factors, and risk of invasive breast cancer stratified by pathologic subtype in the NBCSP case-control study

		Hormone receptor-positive and HER2-negative breast cancer			HER2-positive breast cancer			Hormone receptor-negative and HER2-negative breast cancer	
**Baseline risk factor**	**No. of controls (%)**	**No. of cases (%)**	**Odds ratio^a^(95% CI)**		**No. of cases (%)**	**Odds ratio^a^(95% CI)**		**No. of cases (%)**	**Odds ratio^a^(95% CI)**

Age at first live birth^b^									
5-year increase	4,048 (86.8)	534 (82.0)	1.15 (1.02-1.28)		94 (84.7)	1.13 (0.88-1.44)		57 (79.2)	0.72 (0.49-1.06)
Nulliparous	618 (13.2)	117 (18.0)	1.31 (1.03-1.66)		17 (15.3)	1.04 (0.58-1.86)		15 (20.8)	1.23 (0.63-2.39)
Age at menopause^c^									
5-year increase	3,040 (65.2)	415 (63.8)	1.11 (0.98-1.27)		62 (55.9)	1.36 (0.97-0.69)		50 (69.4)	1.01 (0.68-1.51)
Premenopausal	1,626 (34.8)	236 (36.3)	1.15 (0.87-1.51)		49 (44.1)	1.29 (0.69-2.43)		22 (30.6)	0.88 (0.40-1.92)
Family history of breast cancer									
None	4,055 (86.9)	538 (82.6)	1.00 (reference)		90 (81.1)	1.00 (reference)		54 (75.0)	1.00 (reference)
Second-degree relative	325 (7.0)	66 (10.1)	1.46 (1.09-1.95)		11 (9.9)	1.51 (0.76-3.01)		4 (5.6)	0.67 (0.22-2.01)
First-degree relative ≥ 50 years	172 (3.7)	29 (4.5)	1.13 (0.74-1.73)		4 (3.6)	1.26 (0.43-3.68)		8 (11.1)	2.28 (0.94-5.48)
First-degree relative < 50 years	114 (2.4)	18 (2.8)	1.10 (0.65-1.87)		6 (5.4)	2.82 (1.10-7.20)		6 (8.3)	3.59 (1.25-10.33)
Previous breast biopsy									
No	4,263 (91.4)	547 (84.0)	1.00 (reference)		98 (88.3)	1.00 (reference)		59 (81.9)	1.00
Yes	403 (8.6)	104 (16.0)	1.80 (1.40-1.32)		13 (11.7)	1.39 (0.74-2.60)		13 (18.1)	1.84 (0.90-3.76)
Mammographic density (%)									
0-10	1,710 (36.6)	154 (23.7)	1.00 (reference)		24 (21.6)	1.00 (reference)		16 (22.2)	1.00 (reference)
11-25	1,136 (24.3)	143 (22.0)	1.44 (1.12-1. 85)		27 (24.3)	1.58 (0.88-2.82)		13 (18.1)	1.27 (0.56-2.89)
26-50	1,157 (24.8)	202 (31.0)	2.01 (1.57-2. 57)		42 (37.8)	2.61 (1.47-4.63)		29 (40.3)	3.18 (1.54-6.55)
> 50	663 (14.2)	152 (23.3)	2.60 (1.95-3. 45)		18 (16.2)	1.68 (0.83-3.41)		14 (19.4)	3.15 (1.26-7.84)
*P *value for trend^d^			<0.001			0.024			0.001

^a^Odds ratios and 95% confidence intervals (CIs) for pathologic subtypes of invasive breast cancer obtained from separate multivariate conditional logistic regression models adjusted for all risk factors shown in the table. ^b^Adjusted odds ratios per 5-year increase in age at first live birth among parous women, as well as for nulliparous women compared with women having their first live birth at 25 years. ^c^Adjusted odds ratios per 5-year increase in age at menopause among postmenopausal women, as well as for premenopausal women compared with women having their menopause at 45 years. ^d^*P *values for linear trend using an ordinal variable with values 1 through 4 across successive categories of mammographic density.

## Discussion

In this population-based study of regularly screened women, MD displayed a strong and consistent association with subsequent BC. A high proportion of dense tissue increased the risk of DCIS and invasive BC. MD was associated with screen-detected tumors, but the excess risk was substantially higher for interval cases. Finally, MD proved to be an important risk factor for all pathologic subgroups.

Previous studies have suggested that MD is associated mainly with tumors with hormonal receptors [15-17], whereas others, in agreement with our results, reported a similar or even stronger association with ER^-^/PR^- ^tumors [13,14,18,19]. Overexpression of HER2 was evaluated in a few studies, although their results, like ours, confirm that high MD also increases the risk of HER2^+ ^tumors [13,14,18]. Finally, the two studies that furnished information on triple-negative tumors also found a strong association between MD and this pathologic subtype [13,18].

Risk estimates were particularly high for interval cases. According to our results, interval tumors are 7 times more frequent among women with MD >75% than among those with MD <10%. A previous study in the United States found relative risks similar to those reported here for the highest MD category [11]. Although this strong association can be partly explained by the increased difficulty of tumor detection in highly dense breasts, it may also be related to enhanced proliferation under the stimulus of collagen and stromal growth factors [22]. Indeed, less-favorable subtypes are more frequently found among interval cases [23,24]. In our study, triple-negative and HER^+ ^tumors accounted for 6% and 12% of all screen-detected invasive tumors, respectively, whereas 14% of interval cases were triple negative, and up to 18% were HER2^+^. Interval tumors tend to be more aggressive and have a poorer prognosis [25].

MD is influenced by classic BC risk factors, such as family history, reproductive factors, benign breast disease, and others [5,6,26]. According to our results, however, adjusting for family history and other possible confounders slightly altered the estimated effect. Nevertheless, information on our participants' body mass index (BMI) was not available and could not be taken into account. BMI is a well-established risk factor for postmenopausal BC and is inversely associated with MD, because fat is also stored in the breast, reducing the relative amount of dense tissue. It has been shown that the adjustment for BMI increases the magnitude of the MD effect [27], and that MD reverses the negative association between BMI and BC in premenopausal women [28]. In our case, failure to adjust for BMI would imply an underestimation of the MD effect and explain why relative risks here are smaller than those reported by other studies [3]. This underestimation would be more important in postmenopausal women, and may partly explain why the association between MD and breast cancer was stronger in the premenopausal group. However, a recent meta-analysis reviewing BC risk factors in women in their 40s confirmed that MD is one of the strongest determinants among these women [29]. The underestimation would also be more pronounced for ER-positive tumors, the subgroup that shows a stronger association with BMI [30]. The lack of information regarding alcohol intake may also be considered a limitation. However, its potential effect as a confounder would be very limited in our case, taking into account the patterns of consumption of these women. A previous study in screening participants in Spain showed that 42% of these women did not consume alcohol, and 72% of those who did reported an intake lower than 10 g/day [31]. This study found a modest association between alcohol intake and MD only in postmenopausal women [31], far too small to explain the strong association between MD and BC reported here. In spite of the previously mentioned considerations, the stability of risk estimates in subgroup analyses, the association between MD and all breast cancer subtypes, and the persistence of its effect for at least 7 to 8 years (according to our and others' findings [12]), would support the use of MD as a risk marker to be considered when seeking to adapt screening recommendations.

Mammography screening should maintain a delicate balance between benefit and risk [32]. One in five Spanish women regularly screened would receive a false-positive result, resulting in unnecessary reexaminations and invasive procedures [33]. Overdiagnosis, with ensuing overtreatment, is a particular concern. A recent study has shown that, not only DCIS, but also some invasive screen-detected tumors might spontaneously regress [34]. Conversely, interval tumors are an important indicator of the potential effectiveness of screening. Although interval cases include false-negative results, most of these (65% to 75%) are fast-growing cancers that were not visible in the previous mammogram [23,24,35]. In this respect, it seems important to identify the small group of high-risk women who may benefit from an *ad hoc *follow-up scheme [36]. A recent study in the United States corroborates the fact that, as age or breast density increased, many fewer women needed to be screened to prevent one death of breast cancer [10]. The authors propose different personalized screening schemes according to MD and the presence/absence of established risk factors [10], but this option should be tested before being transferred to on-going screening programs. To maximize the impact on mortality while time avoiding screening side effects, such programs should follow established protocols until new schemes prove to be equally or more effective.

This study, set in the context of a fully consolidated population-based screening program with a high participation rate, confirms the strong relation between MD and BC, regardless of invasiveness, pathologic subtype, and means of diagnosis. To interpret our results, however, some limitations should be borne in mind. First, MD was visually assessed, implying a certain degree of subjectivity. Computer-assisted methods might be a better choice, but they are not exempt from subjectivity, require specific training, and are labor intensive and difficult to incorporate into mass screening [37]. Second, as mentioned earlier, BMI was not available and could not be taken into account, something that translates into an underestimation of the real excess risk associated with MD. Last, tumor classification was based on data registered in the corresponding pathologic records at the two hospitals caring for breast cancer patients in the region. A certain degree of misclassification cannot be ruled out.

## Conclusions

High MD was associated with all pathologic subgroups of breast cancer. The excess risk persisted at least 7 to 8 years after mammographic assessment. Our results confirm that MD is an important risk factor regardless breast cancer subtype and the method of detection. The risk of developing an interval cancer is higher in women with dense breasts, and their excess risk is not explained by a masking effect. The introduction of digital mammographic devices and the development of integrated software to estimate MD will help overcome the drawbacks of currently established methods [37] and facilitate the incorporation of MD assessment in the routine of breast cancer screening.

## Abbreviations

BC: breast cancer; BMI: body mass index; DCIS: ductal carcinoma *in situ*; MD: mammographic density; OR: odds ratio.

## Competing interests

The authors declare that they have no conflicts of interest.

## Authors' contributions

MP, NA, JEAM, and RP conceived the study and participated in its design. NA, ME, and NE collected and checked the information recorded by the screening program, and performed the matching process and the record linkage with the Navarre Breast Cancer Registry. ME and NE collected the pathologic records, and JEAM helped them in the classification process. AM evaluated breast density. MP and RP performed the statistical analysis, and results were circulated and commented on by all the authors. MP, NA, and RP drafted the first version, and it was critically reviewed by the rest of authors. All authors read and approved the final manuscript.

## Acknowledgements

This work was supported by research grants from Eli Lilly and Company (EV1 1082/08); and the Spanish Federation of Breast Cancer Patients (*Federación Española de Cáncer de Mama*) (FECMA 485 EPY 1170-10).

## Supplementary Material

Additional file 1 **Association between mammographic density and other selected risk factors, and risk of total breast cancer stratified by menopausal status**. Results from the multivariate model separately fitted in pre-and postmenopausal women. Click here for file
