# Supplementary material for: Mammographic density and risk of breast cancer according to tumor characteristics and mode of detection: a Spanish population-based case-control study
Source: Breast Cancer Res. 2013 Jan 29;15(1):R9. doi: 10.1186/bcr3380 (PMC3672793; doi:10.1186/bcr3380)
Supplement: Additional file 1 — Association between mammographic density and other selected risk factors, and risk of total breast cancer stratified by menopausal status. Results from the multivariate model separately fitted in pre-and postmenopausal women. [file bcr3380-S1.DOC]

**Table S1.** Association between mammographic density and other selected risk factors, and risk of total breast cancer stratified by menopausal status.

|  | **Postmenopausal** | | | **Premenopausal** | | |
| --- | --- | --- | --- | --- | --- | --- |
| **Baseline risk factor** | **No. of controls (%)** | **No. of cases (%)** | **Odds ratio^a^ (95% CI)** | **No. of controls (%)** | **No. of cases (%)** | **Odds ratio^a^ (95% CI)** |
| Age at first live birth^b^ |  |  |  |  |  |  |
| 5-year increase | 2614 (86.0) | 588 (80.8) | 1.09 (1.00–1.23) | 1434 (88.2) | 352 (82.2) | 1.14 (0.99–1.32) |
| Nulliparous | 426 (14.0) | 140 (19.2) | 1.29 (1.02–1.63) | 192 (11.8) | 76 (17.8) | 1.43 (1.04–1.96) |
| Age at menopause^c^ |  |  |  |  |  |  |
| 5-year increase | 3040 (100) | 728 (100) | 1.16 (1.04–1.28) |  |  |  |
| Family history of breast cancer |  |  |  |  |  |  |
| None | 2650 (87.2) | 600 (82.4) | 1.00 (reference) | 1405 (86.4) | 332 (77.6) | 1.00 (reference) |
| Second-degree relative | 194 (6.4) | 56 (7.7) | 1.24 (0.90–1.71) | 131 (8.1) | 51 (11.9) | 1.62 (1.11–2.36) |
| First-degree relative ≥ 50 years | 117 (3.9) | 43 (5.9) | 1.42 (0.97–2.07) | 55 (3.4) | 21 (4.9) | 1.70 (0.97–2.98) |
| First-degree relative < 50 years | 79 (2.6) | 29 (4.0) | 1.52 (0.97–2.41) | 35 (2.2) | 24 (5.6) | 2.74 (1.52–4.93) |
| Previous breast biopsy |  |  |  |  |  |  |
| No | 2780 (91.5) | 621 (85.3) | 1.00 (reference) | 1483 (91.2%) | 365 (85.3) | 1.00 (reference) |
| Yes | 260 (8.6) | 107 (14.7) | 1.63 (1.26–2.11) | 143 (8.8%) | 63 (14.7) | 1.49 (1.05–2.10) |
| Mammographic density (%) |  |  |  |  |  |  |
| 0–10 | 1420 (46.7) | 240 (33.0) | 1.00 (reference) | 290 (17.8) | 23 (5.4) | 1.00 (reference) |
| 11–25 | 758 (24.9) | 181 (24.9) | 1.42 (1.14–1.77) | 378 (23.3) | 63 (14.7) | 2.01 (1.19–3.39) |
| 26–50 | 597 (19.6) | 210 (28.9) | 2.09 (1.67–2.62) | 560 (34.4) | 165 (38.6) | 3.50 (2.17–5.65) |
| 51–75 | 217 (7.1) | 80 (11.0) | 2.10 (1.52–2.89) | 311 (19.1) | 135 (31.5) | 4.88 (2.97–8.01) |
| > 75 | 48 (1.6) | 17 (2.3) | 1.98 (1.04–3.76) | 87 (5.4) | 42 (9.8) | 5.13 (2.83–9.30) |
| *P* value for trend^d^ |  |  | < 0.001 |  |  | < 0.001 |

^a^ Odds ratios and 95% confidence intervals (CIs) for screen-detected and interval breast cancer obtained from separate multivariate conditional logistic regression models adjusted for all risk factors shown in the table.

^b^ Adjusted odds ratios per 5-year increase in age at first live birth among parous women, as well as for nulliparous women compared to women having their first live birth at 25 years.

^c^ Adjusted odds ratios per 5-year increase in age at menopause among postmenopausal women, as well as for premenopausal women compared to women having their menopause at 45 years.

^d^ *P* values for linear trend using an ordinal variable with values 1 through 6 across successive categories of mammographic density.
